# Dexamethasone potentiates the antiangiogenic activity of docetaxel in castration-resistant prostate cancer

**DOI:** 10.1038/sj.bjc.6604804

**Published:** 2008-12-02

**Authors:** C Wilson, P Scullin, J Worthington, A Seaton, P Maxwell, D O'Rourke, P G Johnston, S R McKeown, R H Wilson, J M O'Sullivan, D J J Waugh

**Affiliations:** 1 Centre for Cancer Research and Cell Biology, Queen's University Belfast, Belfast BT9 7BL, Northern Ireland; 2 School of Biomedical Sciences, University of Ulster, Coleraine BT52 1SA, Northern Ireland; 3 Department of Histopathology, Belfast City Hospital, Belfast BT9 7AB, Northern Ireland

**Keywords:** dexamethasone, docetaxel, angiogenesis, prostate cancer

## Abstract

We sought to characterise whether dexamethasone (DEX) may enhance tumour response to docetaxel in *in vitro* and *in vivo* models of metastatic prostate cancer (CaP). *In vitro* experiments conducted on PC3 and human bone marrow endothelial cells (hBMECs) determined that administration of DEX (10 nM) reduced constitutive nuclear factor-*κ*B (NF-*κ*B) activity, decreasing interleukin (IL)-8, CXCL1 and *VEGF* gene expression in PC3 cells. Dexamethasone also attenuated docetaxel-induced NF-*κ*B and activator protein-1 transcription and reduced docetaxel-promoted expression/secretion of IL-8 and CXCL1 in PC3 and hBMECs. Although DEX failed to enhance docetaxel cytotoxicity on PC3 cells, DEX potentiated the antiangiogenic activity of docetaxel *in vitro*, further reducing vessel area and vessel length in developing endothelial tubes (*P*<0.05). Docetaxel had a potent antiangiogenic activity in the dorsal skin flap-implanted PC3 tumours *in vivo*. Small blood vessel formation was further suppressed in tumours co-treated with docetaxel and DEX, substantiated by an increased average vessel diameter and segment length and a decreased number of branch points in the residual tumour vasculature (*P*<0.001). Our data show that DEX potentiates the antiangiogenic activity of docetaxel, suggesting a putative mechanism for the palliative and survival benefits of these agents in metastatic CaP.

Prostate cancer (CaP) is now the second most common cause of male cancer death in the Western society. Castration therapy (either surgical orchidectomy or luteinizing hormone releasing hormone agonists) is initially effective in treating recurrent or metastatic CaP. However, the majority of these patients will become refractory to castration as the cancer loses dependence on androgen-signalling. As a further complication, castration-resistant CaP (CRPC) is poorly sensitive to conventional cytotoxic chemotherapy. However, two recent phase III trials, the TAX 327 and SWOG 9916 trials, have reported a survival benefit together with superior pain response, PSA response rate and improvement in quality of life in patients with hormone refractory, metastatic CaP receiving docetaxel (Taxotere^-^) as opposed to mitoxantrone therapy. Accordingly, docetaxel has become the chemotherapy of choice for men with metastatic CRPC (Petrylak *et al*, 2004; Tannock *et al*, 2004).

Glucocorticoids, such as dexamethasone (DEX), have also been shown to exhibit single-agent activity in CRPC. Low dose, daily DEX administration has been reported to yield biochemical PSA response rates of up to 49% in CRPC (Venkitaraman *et al*, 2008). The mechanism by which glucocorticoids exert their effect in cancer is not well elucidated, although several mechanisms have been postulated. Glucocorticoids have been shown to interfere with the transcriptional activity of nuclear factor-*κ*B (NF-*κ*B) and activator protein-1 (AP-1) (Nishimura *et al*, 2001), resulting in the suppression of the interleukin-6 (IL-6) and the NF-*κ*B–IL-6 pathways in CaP (Akakura *et al*, 2003; De Bosscher *et al*, 2003). Dexamethasone has also been shown to inhibit angiogenesis in a CaP xenograft model, reducing cell and tumour IL-8 and VEGF expression in *in vitro* and *in vivo* assays, respectively (Yano *et al*, 2006).

CXC-chemokines including IL-8 have an established role in promoting the progression of CaP (Waugh *et al*, 2008). Interleukin-8 expression has been shown to be elevated in CaP tissue by immunohistochemistry (Huang *et al*, 2005; Murphy *et al*, 2005) and by *in situ* hybridisation (Uehara *et al*, 2005), whereas overexpression of IL-8 has been detected in the serum of CaP patients (Veltri *et al*, 1999; McCarron *et al*, 2002). Furthermore, IL-8 expression in CaP tissue has been associated with increased biochemical recurrence (Caruso *et al*, 2008). Consistent with this, studies in athymic mice have correlated the increased expression of IL-8 in implanted human CaP cells with increased vascularisation of the tumours and an enhanced tumourigenic and metastatic potential (Inoue *et al*, 2000; Kim *et al*, 2001). Other studies have determined the relevance of IL-8 signalling to the development of androgen-independence (Araki *et al*, 2007; Seaton *et al*, 2008), CaP cell proliferation (Murphy *et al*, 2005) and CaP cell survival in response to environmental and chemotherapy/biological agents (Maxwell *et al*, 2007; Wilson *et al*, 2008a, 2008b).

Glucocorticoids are frequently provided to patients to offset fluid retention and hypersensitivity responses to taxane administration and were present as an element of the TAX327 and SWOG9916 trial regimens. Furthermore, given that DEX exhibits single-agent activity in this disease (Venkitaraman *et al*, 2008), we established this pre-clinical study to determine whether two agents used clinically and often together during therapy may act to enhance the activity of the other in experimental models of CRPC. Therefore, our experiments focused on elucidating the effect of DEX and docetaxel in modulating CXC-chemokine expression and signalling within CRPC cells, with the intent of characterising a mechanism to explain how DEX might enhance docetaxel response in this disease.

## Materials and methods

### Chemical and reagents

Chemicals were purchased from Sigma Chemical Co. (St Louis, MO, USA) unless otherwise stated.

### Cell culture

Human PC3 and LNCaP cells were sourced and grown in RPMI 1640 medium supplemented with 10% fetal bovine serum and 4 mM L-glutamine (Invitrogen Ltd, Paisley, UK) as previously described (Maxwell *et al*, 2007; Wilson *et al*, 2008a). The hBMEC line was kindly provided by Dr Babette B Weksler of Weill Medical College, Cornell University, New York. Human BMECs were maintained in DMEM medium supplemented with 5% FCS, 3% L-glutamine and HEPES (10 mM). All cultures were maintained in a humidified chamber at 37°C with 5% CO_2_.

### Cell count analysis

Cells were seeded into 24-well plates (1 × 10^5^ cells per well) in RPMI 1640 medium and allowed to attach overnight. Cells were then treated with DEX (10 nM). Plates were incubated in a humidified chamber at 37°C with 5% CO_2_ for 24, 48 or 72 h, and the cells were trypsinised and counted in triplicate using a Coulter Z series particle count and size analyzer (Beckman Coulter, High Wycombe, UK). Cell numbers were normalised to control values.

### Quantitative real-time PCR (QPCR)

Total RNA was isolated using RNAStat60 (Biogenesis, Oxford, UK) according to the manufacturer's instructions. For real-time PCR, 10 *μ*g of total RNA was oligo(dT) reverse transcribed using MMLV-RT (Invitrogen) according to the manufacturer's instructions. The cDNA (100 ng) was mixed with primers (2 *μ*M), sterile water and SYBR Green PCR mastermix (Finnzymes, Espoo, Finland). The primer sequences were as follows: 18S: forward, 5′-CAT TCg TAT TGC GCC gCT-3′; reverse, 5′- CGA Cgg TAT CTg ATC gTC-3′; IL-8: forward, 5′-ATg ACT TCC AAg CTg gCC gTg g; reverse, 5′- CAT AAT TTC TGT GTT ggC gCA gTg Tgg; CXCL1: forward, CCC AAg AAC ATC CAA AgT gTC A; reverse, gTg gCT ATg ACT TCg gTT Tg; VEGF: forward, 5′- AgC TAC TgC CAT CCA ATC gA; reverse, 5′- ggT gAg gTT TgA TCC gCA TA. Real-time PCR was carried out in a 96-well plate using an Opticon 2 Continuous Fluorescence Detector (Biorad, Hertfordshire, UK). Amplification was 95°C for 15 min, and 45 cycles at 95°C for 15 s, 55°C for 30 s and 72°C for 60 s. The threshold cycle (*C*_t_), which indicates the relative abundance of a particular transcript, was calculated for each reaction by the Opticon 2 system. Expression levels were determined from standard curve dilutions and normalised for 18 s.

### Transfection and luciferase assay

Cells were plated in six-well plates (1 × 10^5^ cells per well) in RPMI 1640 medium and incubated for 48 h at 37°C with 5% CO_2_. Cells were transfected using GeneJuice transfection reagent (Merck Chemicals. Nottingham, UK), according to manufacturer's protocol, with 2 *μ*g pGL3 basic vector (Promega, Madison, WI, USA) or 2 *μ*g of an NF-*κ*B-LUC plasmid (kindly provided by Dr James Purcell, QUB) or 0.5 *μ*g of an AP-1-LUC plasmid (kindly provided by Dr Massimo Gadina, QUB). Cells were also co-transfected with 0.2 *μ*g of a *Renilla* luciferase plasmid as a transfection control for pGL3 and NF-*κ*B or 0.01 *μ*g of a *Renilla* luciferase plasmid as a transfection control for AP-1. Cells were incubated for 24 h before drug addition. The drug of interest was added for the desired time and the samples were analysed by luciferase assay using the Promega Dual Luciferase assay kit (Promega) according to the manufacturer's protocol.

### Enzyme-linked immunosorbent assay (ELISA)

Cells were plated in 24-well plates (5 × 10^4^ cells per well) in RPMI 1640 medium. Following an overnight incubation, the medium was replaced with fresh media, docetaxel (1 nM) or DEX (10 nM), or a combination of both drugs were added to cells for the indicated time. A time-matched control with no drug was also included. The supernatant was removed from the cells and centrifuged at 1000 **g** for 5 min to remove any cell debris and the supernatant was stored at −20°C until assayed. The cell number in each well was determined by parallel cell count analysis. CXCL8 levels were measured using the Pelikine Compact™ IL-8 ELISA Kit (Sanquin Reagents, Amsterdam, The Netherlands), whereas CXCL1 levels were determined using the Quantikine® kit (R&D Systems, Abingdon, UK). The manufacturer's instructions were used in the application of each ELISA kit. Absorbance readings were taken at 450 nm using a microwell plate reader (Molecular Devices, Wokingham, UK).

### 3-(4,5-dimethylthiazol-2-yl)-2,5-diphenyltetrazolium bromide (MTT) assay

Cells were seeded into 96-well plates (3 × 10^3^ cells per well) in RPMI 1640 medium and allowed to attach overnight. Serial dilutions of docetaxel (kind gift from Belfast City Hospital Pharmacy) or DEX were added to the cells alone and, in the case of docetaxel, in combination with a fixed concentration of DEX (10 nM). In other experiments, cells were treated with 5 *μ*M BAY-11-7082 (Calbiochem, La Jolla, CA, USA), a monoclonal neutralising antibody for IL-8, at a final concentration of 4 *μ*g ml^−1^ (R&D Systems, Abingdon, UK) or a selective CXCR2 receptor antagonist AZ10397767 (Walters *et al*, 2008) (kindly provided by Dr Simon T. Barry, AstraZeneca, Alderley Park, UK). Plates were incubated in a humidified chamber at 37°C with 5% CO_2_ for 72 h, then 50 *μ*l MTT (2 mg ml^−1^) was added and the plates returned to the incubator for 4 h. Medium and any unmetabolised MTT was aspirated from the wells and the formazan crystals were dissolved in 100 *μ*l dimethyl sulphoxide. Absorbance was read at 570 nm using a microplate reader (Molecular Devices).

### Angiogenesis assay

AngioKit (GB patent 2331763) consisting of one 24-well pre-seeded tissue culture plate containing early stage co-cultures of human endothelial cells and other human cells, five 25 ml single use bottles of optimised media, positive (VEGF) and negative (suramin) controls and CD31 (PECAM-1) staining kit was supplied by TCS CellWorks Ltd (Buckingham, UK). Fresh media, media with positive control (VEGF), media with suramin or media plus docetaxel (1 nM), DEX (10 nM) or a combination of both was added to each well on days 1, 4, 7 and 9 as per the manufacturer's instructions. Cells were treated with each test compound in triplicate. Plates were incubated in a humidified chamber at 37°C with 5% CO_2_ for 11 days, then fixed and stained with anti-CD31 according to the manufacturer's instructions. Images were analysed using a LEICA DMLB microscope and LUICA measurement software (Leica Microsystems (UK) Ltd, Milton Keynes, UK). For each well, 15 fields were analysed using the five-times objective. The length and area of each vessel were measured using the LUICA measurement software. Mean vessel length and area, as well as total vessel length and area in 15 fields in each of the triplicate wells were calculated (Microsoft Excel software).

### Dorsal skin flap model

Imaging of the tumour vasculature in an implanted PC3 tumour was conducted in 12-week-old male BALB-c SCID mice as previously described (O’Rourke *et al*, 2008). Briefly, a viewing chamber was attached to a raised skin flap on the dorsal flank of the mouse under anaesthesia and a fragment (0.5 mm) of PC3 prostate tumour (excised from a donor mouse) was placed on the exposed microvascular bed. The PC3 tumour vasculature was imaged 7 days postsurgery, (tumours measured 2 mm in diameter), by injection of 50 *μ*l of 150 kDa FITC-labeled dextran (50 mg ml^−1^) into the tail vein under light anaesthesia. The viewing chamber was illuminated with a mercury lamp, and the FITC signal was detected with an ORCA-ER camera system (Hamamatsu Photonics, Welwyn Garden City, UK). Images were recorded using Image J software (NIH, Bethesda, MD, USA). Mice then received DEX (10 nM), docetaxel (0.5 nM) or a combination of DEX (10 nM)/docetaxel (0.5 nM), administered directly to the sealed chamber on days 8, 11 and 14 post-implantation. Control animals received injections of PBS at similar time points. Imaging was repeated at days 9, 11, 15 and 18 post-implantation. Experiments were carried out in accordance with the Animal (Scientific Procedures) Act 1986 and conformed to the current United Kingdom Coordinating Committee on Cancer Research guidelines.

### Statistical analysis

Two-tailed Student's *t*-tests were performed using GraphPad Prism 4.0 software to compare mean values in drug-treated CaP and endothelial cells for each of the following *in vitro* experiments: analysis of gene expression and secretion, comparison of cell viability and determination of vessel formation in the AngioKit assay. Statistical analysis of the tumour vascular parameters in the dorsal skin flap model was performed using a one-way ANOVA (Microsoft Excel software).

## Results

### Effect of DEX on basal NF-*κ*B activity in PC3 cells

**Figure 1:**
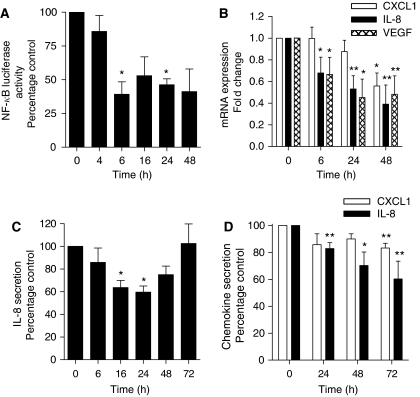
Dexamethasone (DEX) decreases expression and secretion of proangiogenic CXC-chemokines in PC3 cells. (**A**) Bar graph illustrating the effect of 10 nM DEX on NF-*κ*B-driven transcriptional activity in PC3 cells transfected with a pGL3-NF-*κ*B-LUC plasmid compared with time-matched controls transfected with an empty vector and normalised against *Renilla* luciferase activity as described in Materials and Methods. Data shown are the mean±s.e.m. of six independent experiments. (**B**) Bar graph illustrating the relative mRNA transcript levels for the proangiogenic factors IL-8, CXCL1 and VEGF in PC3 cells, determined by QPCR over a 48 h time course post-treatment with 10 nM DEX. Values shown represent the mean±s.e.m. value, determined from three or four independent experiments. (**C**) Bar graph illustrating the relative secretion of the CXC-chemokine IL-8 from PC3 cells following treatment with a single administration of 10 nM DEX. The concentration of IL-8 secreted into the culture media was determined by ELISA. Data shown are the mean±s.e.m. value of six independent experiments. (**D**) As in (**C**), except that 10 nM DEX was administered to the PC3 cells every 24 h. Data shown are the mean±s.e.m. value of four independent experiments. Statistically significant differences in activity, transcript levels or secretion were determined using a two-tailed Student's *t*-test analysis. ^*^P<0.05; ^**^P<0.01.

Constitutive activation of the NF-*κ*B transcription factor has been reported in CaP and is associated with disease progression, chemoresistance, angiogenesis and metastasis (Huang *et al*, 2001; Fradet *et al*, 2004; Ross *et al*, 2004; Shukla *et al*, 2004; Sweeney *et al*, 2004; Domingo-Domenech *et al*, 2005). Nuclear factor-*κ*B has been reported to induce the transcription of proinflammatory genes, many of which have been implicated in each of the above phenotypes. Given the anti-inflammatory activity of glucocorticoids, we initially determined the effect of DEX administration upon the constitutive transcriptional activity of NF-*κ*B in PC3 cells. The PC3 cells were transfected with the pGL3-NF-*κ*B luciferase construct. Addition of DEX (10 nM) was shown to effect a sustained repression of NF-*κ*B-luciferase activity in transfected PC3 cells. The maximal inhibition of NF-*κ*B activity was observed 6 h post-administration of DEX, attenuating NF-*κ*B activity to 39% of that in control cells (*P*<0.05) (Figure 1A).

### DEX downregulates IL-8 and VEGF expression in PC3 cells

Having shown that DEX downregulates NF-*κ*B activity, we confirmed whether DEX modulated the expression of the proangiogenic NF-*κ*B transcriptional target, IL-8. Using QPCR, we observed a time-dependent decrease in IL-8 transcript levels in PC3 cells following a single administration of DEX (10 nM), suppressing the constitutive IL-8 mRNA transcript expression in PC3 cells to 38% of that in untreated cells 48 h post-treatment with DEX (*P*<0.01) (Figure 1B). Similarly, administration of DEX was also shown to reduce the expression of the related proangiogenic CXC-chemokine, CXCL1 (*P*<0.05 at the 48 h time point). In a further analysis, we determined the effect of DEX on the gene expression of VEGF, another angiogenesis-promoting factor whose transcription is also regulated by NF-*κ*B activity. The VEGF mRNA transcript levels also showed a time-dependent decrease following administration of DEX (10 nM) to PC3 cells that was evident within 6 h and was maximal (45%) by 24 h (Figure 1B).

To confirm that the observed downregulation of IL-8 mRNA transcript levels in PC3 cells corresponded with a decreased rate of IL-8 secretion, we performed an ELISA. The PC3 cells were treated with a single dose of DEX (10 nM), and media samples were collected between 6 and 72 h post-administration of the glucocorticoid. To account for any time-dependent accumulation of secreted IL-8 in cultured PC3 cells, we collected a time-matched control sample alongside each treated sample. The secretion of IL-8 was normalised relative to cell number and expressed as a percentage of the time-matched, untreated sample. Administration of DEX (10 nM) effected a time-dependent suppression of IL-8 secretion from PC3 cells, reaching a maximal inhibition at 24 h (*P*<0.05). However, the level of IL-8 secretion recovered to baseline levels 72 h post-treatment (Figure 1C). Similarly, experiments with DEX effected a similar reduction in basal IL-8 secretion from androgen-dependent CaP cells (LNCaP), reducing the secretion to 75% of basal levels 24 h after addition of DEX (*P*<0.05) (data not shown). As in the clinical setting, DEX is administered daily, we also determined the effect of daily DEX (10 nM) on IL-8 and CXCL1 secretion in PC3 cells; this prevented the return to basal levels of IL-8 secretion and also resulted in a sustained, although smaller, decrease in CXCL1 secretion (Figure 1D).

### Effect of DEX on PC3 and endothelial cell proliferation

Experiments were established to determine the effect of DEX on PC3 cell proliferation. The PC3 cells were treated with DEX over a concentration range of 0.1 nM to 10 *μ*M for 72 h and an MTT assay performed to assess cell viability. Dexamethasone had no inhibitory effect on PC3 cell proliferation at any concentration studied over this 72 h period (data not shown). The effect of DEX on PC3 cell growth over a time course was also examined. Cell count analysis was performed at 24, 48 and 72 h after addition of 10 nM DEX to PC3 cells. Relative to unstimulated cells, administration of 10 nM DEX was observed to have no significant effect on cell proliferation (data not shown). Dexamethasone (0–1 *μ*M) also had no inhibitory effect on LNCaP or hBMEC cell proliferation (data not shown).

### DEX attenuates docetaxel-induced NF-*κ*B and AP-1 activity in PC3 cells

**Figure 2:**
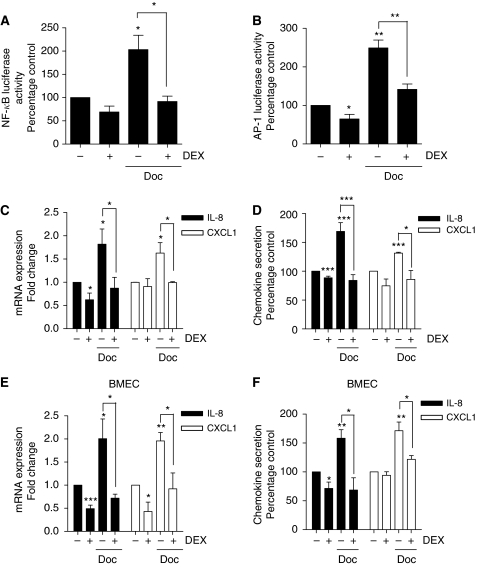
Effect of dexamethasone (DEX) upon docetaxel-induced transcriptional regulation and potentiation of CXC-chemokine expression/secretion by PC3 and endothelial cells. (**A**) Bar graph illustrating the effect of 1 nM docetaxel (Doc) or 10 nM DEX, singly or in combination, upon NF-*κ*B-driven transcriptional activity in PC3 cells transfected with a pGL3-NF-*κ*B-LUC plasmid measured 24 h post-addition of the drugs. This is compared with time-matched controls transfected with an empty vector and normalised against *Renilla* luciferase activity as described in Materials and Methods. Data shown are the mean±s.e.m. of four independent experiments. (**B**) Bar graph illustrating the effect of 1 nM Doc or 10 nM DEX, singly or in combination, upon AP-1-driven transcriptional activity in PC3 cells transfected with a pGL3-AP-1-LUC plasmid measured 24 h post-administration of the drugs. This is compared with time-matched controls transfected with an empty vector and normalised against *Renilla* luciferase activity as described in Materials and Methods. Data shown are the mean±s.e.m. of four independent experiments. (**C**) Bar graph illustrating the relative mRNA transcript levels for the proangiogenic factors IL-8 and CXCL1 in PC3 cells 24 h post-treatment with 1 nM Doc or 10 nM DEX, administered singly or in combination. Transcript levels were determined using the established QPCR protocols. Values shown represent the mean±s.e.m. value of four independent experiments. (**D**) Bar graph illustrating the levels of CXC-chemokine secretion from PC3 cells 24 h post-treatment with 1 nM Doc or 10 nM DEX, administered singly or in combination and calculated from three independent experiments. (**E** and **F**) As in (**B** and **D**), respectively, except that experiments were conducted in hBMECs. Statistically significant differences in activity, transcript levels or secretion were determined using a two-tailed Student's *t*-test analysis. ^*^P<0.05; ^**^P<0.01; ^***^P<0.001.

Patients with CRPC frequently receive DEX in combination with docetaxel. Using the aforementioned luciferase reporter assays, we studied the impact of administering DEX and docetaxel in combination upon NF-*κ*B and AP-1 transcriptional activity in PC3 cells. Measurements of transcription factor activity were conducted 24 h post-administration of the drug treatments. The PC3 cells were treated with a single dose of docetaxel (1 nM), alone or co-administered with DEX (10 nM). Docetaxel doubled NF-*κ*B activity in the PC3 cells compared with control unstimulated cells (*P*<0.05); however, this was reduced to basal levels in the presence of DEX (*P*<0.05 relative to docetaxel alone) (Figure 2A). Similarly, docetaxel administration increased AP-1 transcriptional activity in PC3 cells by over two-fold (*P*<0.01). Co-administration of DEX attenuated but did not abrogate the docetaxel-induced AP-1 activity in these PC3 cells (*P*<0.01) (Figure 2B).

Having shown that DEX attenuated docetaxel-induced potentiation of NF-*κ*B and AP-1 transcriptional activity, we confirmed the effect of administering these drugs alone or in combination upon the regulation of the IL-8 and CXCL1 genes. The PC3 cells were treated with docetaxel (1 nM) alone, or concurrently with DEX (10 nM). The mRNA samples were harvested from the cells 24 h post-treatment. The mRNA transcript levels for IL-8 and CXCL1 were then quantified relative to that of the housekeeping gene 18S using the established, gene-specific QPCR assays. Docetaxel administration resulted in increasing IL-8 mRNA and CXCL1 mRNA transcript levels to over 1.5-fold of that detected in time-matched, untreated cell populations (Figure 2C); this was attenuated by the concurrent administration of DEX (*P*<0.05 in both cases).

We also used an ELISA to determine the impact of docetaxel on IL-8 and CXCL1 secretion from PC3 cells. All values for CXC-chemokine secretion were normalised against cell number and expressed as a percentage of that measured in time-matched, untreated samples. In initial experiments, we conducted a time course to determine how docetaxel influenced the rate and magnitude of IL-8 secretion from PC3 cells. Docetaxel potentiated IL-8 secretion in a time-dependent manner, increasing the extracellular level of this chemokine to 175 and 160% of that determined in untreated, time-matched controls, 16 and 24 h post-addition of this taxane, respectively (*P*<0.05) (data not shown). In further experiments, the level of IL-8 in the cell media was quantified and normalised 24 h post-administration of docetaxel, in the absence and presence of DEX (10 nM). Docetaxel induced IL-8 secretion to 168% of that detected in untreated PC3 cells (*P*<0.001). However, concurrent treatment with DEX attenuated the docetaxel-induced increase in IL-8 secretion, reducing IL-8 secretion to approximately 10% below that observed in untreated cells (*P*<0.001) (Figure 2D). A similar trend was determined for the effect of these drugs in promoting CXCL1 secretion from PC3 cells. Again, DEX was shown to reverse docetaxel-induced CXCL1 secretion (*P*<0.05) (Figure 2D).

### DEX attenuates docetaxel-induced IL-8 expression and secretion in endothelial cells

Further experiments were conducted on hBMECs to determine the effects of DEX, docetaxel and a combination of DEX with docetaxel on IL-8 and CXCL1 gene transcription and the secretion of these CXC-chemokines by endothelial rather than tumour cells. Experiments were conducted as previously described for the tumour cells. As observed before, addition of DEX to these endothelial cells reduced IL-8 transcript levels to 75% of that observed in untreated, time-matched control cells. Administration of docetaxel (1 nM) was shown to increase IL-8 mRNA transcript levels to the levels approaching double that of control levels, whereas co-treatment with DEX reduced IL-8 transcript levels to 73% of that observed in untreated, time-matched control cells (*P*<0.05) (Figure 2E). Similarly, DEX was shown to reverse docetaxel-induced CXCL1 mRNA transcript levels in these endothelial cells (*P*<0.05) (Figure 2E). Furthermore, using ELISAs, we were able to confirm that co-administration of DEX attenuated the docetaxel-induced increase in IL-8 and CXCL1 secretion from hBMECs (*P*<0.05 in either case) (Figure 2F).

### Effect of DEX administration upon docetaxel cytotoxicity in CRPC and endothelial cells

**Figure 3:**
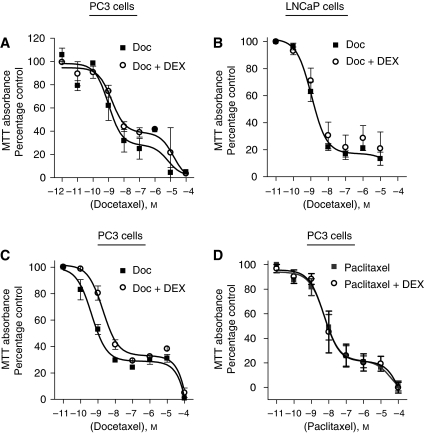
Effect of dexamethasone (DEX) upon the cytotoxicity of taxanes to CaP cells. Graphs showing the effect of docetaxel, in the absence and presence of 10 nM DEX, upon the viability of (**A**) PC3 and (**B**) LNCaP cells. Data points shown on the graphs represent the mean±s.e.m. value calculated from four and three experiments, respectively. (**C**) Graph showing the effect of docetaxel, following pre-treatment (48 h) of PC3 cells with 10 nM DEX. The data points shown represent the mean±s.e.m. of three independent experiments. (**D**) Graph showing the effect of paclitaxel in the absence and presence of 10 nM DEX upon the viability of PC3 cells. The data points shown represent the mean±s.e.m. of three independent experiments. In all scenarios, DEX failed to enhance the cytotoxicity of taxanes on CaP cells.

Nuclear factor-*κ*B and AP-1 transcription are each associated with promotion of cell survival and cell proliferation. We therefore examined the cytotoxicity of docetaxel (0.01 nM to 100 *μ*M) in PC3 cells for 72 h, when administered alone or in combination with DEX (10 nM). An MTT assay was performed to assess cell viability. Concentration-response curves, modelling to a two-site competition equation were generated, suggesting two different modes of action of docetaxel activity in PC3 cells. A concentration-dependent decrease in cell viability was observed following treatment with docetaxel with a calculated IC_50_ value of 2.17 nM. However, concurrent administration of DEX (10 nM) failed to enhance the cytotoxicity of docetaxel (IC_50_ 3.31 nM) or indeed, alter the distribution of viable cells across the two-distinct populations (Figure 3A). Similarly, concurrent administration of DEX did not influence the cytotoxicity of docetaxel in hBMECs (data not shown).

Clinically, DEX is given to patients in the 24 h before docetaxel. As concurrent treatment of DEX (10 nM) failed to alter the potency of docetaxel, we conducted a further series of experiments examining the effect of pre-treating PC3 cells with DEX before docetaxel administration. The PC3 cells were treated with DEX (10 nM) for 48 h, followed by treatment with docetaxel over a concentration range of 0.01 nM to 100 *μ*M for 72 h. Control samples were not pre-treated with DEX. Cell viability was assessed by performing MTT assays. However, pre-treatment with DEX was observed to decrease the cytotoxicity of docetaxel by a factor of five-fold (IC_50_ 5.94 nM), as compared to control (1.2 nM). Therefore, scheduling has no apparent effect on the ability of DEX to increase docetaxel cytotoxicity in CRPC cells (Figure 3C). Experiments were also conducted using another taxane, paclitaxel. Co-administration of DEX similarly failed to increase the potency of paclitaxel in PC3 cells (Figure 3D).

### Suppression of NF-*κ*B activation or extracellular IL-8 levels does not enhance docetaxel-induced cytotoxicity in PC3 cells

**Figure 4:**
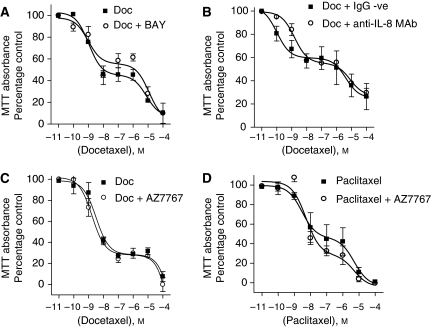
Effect of inhibiting NF-*κ*B, IL-8 or CXC-chemokine expression upon taxane cytotoxicity to PC3 cells. Graphs presenting the effect of co-administering (**A**) 5 *μ*M BAY-11-7082, (**B**) 4 *μ*g ml^−1^ anti-IL-8 monoclonal Ab, or (**C**) 20 nM AZ10397767 upon the cytotoxicity of docetaxel in PC3 cells. (**D**) Graph presenting the effect of administering increasing concentrations of paclitaxel in the absence and presence of 20 nM AZ10397767 upon the viability of PC3 cells. Data points shown represent the mean±s.e.m. of three or four independent experiments. In all scenarios presented in this figure, pharmacological interventions inhibiting the NF-*κ*B pathway or proangiogenic CXC-chemokine signalling failed to enhance the cytotoxicity of taxanes on PC3 cells.

To determine whether a more potent and direct inhibition of NF-*κ*B activity may potentiate the cytotoxicity of docetaxel, PC3 cells were treated with docetaxel in the absence and presence of the NF-*κ*B inhibitor, BAY11-7082, administered at a final concentration of 5 *μ*M. However, BAY11-7082 failed to potentiate the cytotoxicity of docetaxel upon PC3 cells (Figure 4A). We also determined whether the potentiation of IL-8 signalling in response to docetaxel may assist in enabling PC3 cells to withstand taxane therapy. Interleukin-8 signalling was initially inhibited using a neutralising anti-IL-8 monoclonal antibody, administered at a concentration of 4 *μ*g ml^−1^. However, no potentiation of docetaxel sensitivity was observed following the inhibition of extracellular IL-8 levels in the PC3 cell media (Figure 4B). In a further series of experiments, we used the CXCR2 antagonist AZ10397767 at a concentration of 20 nM to attenuate any potential signalling effect resulting from the activity of additional, closely related CXC-chemokines to IL-8 (e.g., CXCL1). However, co-administration of AZ10397767 also had no effect on the cytotoxicity of docetaxel (Figure 4C) or paclitaxel (Figure 4D) in the PC3 cells. Therefore, direct (BAY11-7082) or indirect (DEX) perturbation of the NF-*κ*B transcription factor or two of its downstream signalling effectors (IL-8/CXCR2) does not appear to sensitise PC3 cells to the effects of docetaxel.

### DEX potentiates the antiangiogenic activity of docetaxel

**Figure 5:**
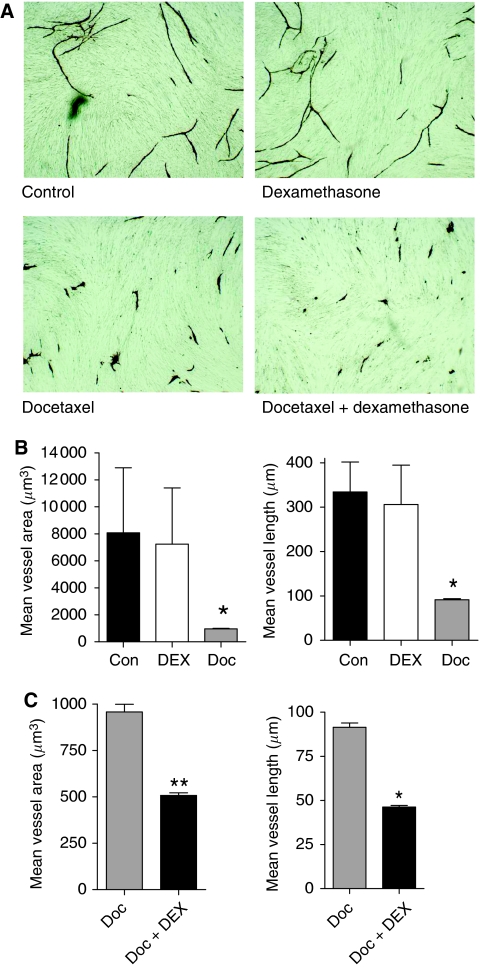
Enhancement of the antiangiogenic effect of docetaxel by combination with dexamethasone (DEX) in endothelial tube formation assays. (**A**) Representative field images of endothelial tube formation following administration of a vehicle control (top left quadrant), 10 nM DEX (top right quadrant), 1 nM docetaxel (Doc) (lower right quadrant) and 1 nM docetaxel co-administered with 10 nM DEX (lower right quadrant). All images shown are at a magnification of × 10. (**B**) Quantitation of mean vessel area (*μ*m^3^) (left panel) and mean vessel length (*μ*M) (right panel) as determined from analysis of 10 representative fields in vehicle control-, 10 nM DEX- or 1 nM Doc-treated endothelial cells. Columns represent the mean±s.e.m. value. (**C**) Comparison of the mean vessel area (*μ*m^3^) (left panel) and mean vessel length (*μ*M) (right panel) as determined by image analysis of 10 representative fields in wells receiving 1 nM Doc-treated *vs* 1 nM Doc plus 10 nM DEX. Columns represent the mean±s.e.m. value. Statistically significant differences in vessel area and length in (**B**) and (**C**) were determined using a two-tailed Student's *t*-test analysis. ^*^P<0.05; ^**^P<0.01.

Dexamethasone and docetaxel are independently associated with the promotion of antiangiogenic effects in *in vivo* models of cancer. Therefore, in the absence of DEX modulating the cytotoxicity of docetaxel to CRPC cells, we investigated whether the capacity of DEX to attenuate the docetaxel-induced expression of proangiogenic factors may enhance the established antiangiogenic activity of taxanes. Initially, we used a commercial AngioKit assay (TCS), which reproduces the different phases of angiogenesis. Docetaxel (1 nM) and/or DEX (10 nM) were added to the AngioKit wells every 72 h and the assay was conducted for 11 days before staining with CD31 as described in Materials and Methods. Administration of DEX alone had a minimal but not statistically significant effect upon either mean vessel area or mean vessel length (Figure 5B). Docetaxel, in contrast, had a marked effect on vessel development resulting in an almost 90% reduction in mean vessel area (*P*<0.05) and an over 70% reduction in mean vessel length compared with untreated cells (*P*<0.05) (Figure 5B). Interestingly, co-administration of DEX was observed to potentiate the antiangiogenic activity of docetaxel. The mean vessel area was reduced by a further 50% in cells treated with DEX and docetaxel compared with the effect of docetaxel alone (*P*<0.01). Similarly, mean vessel length was also reduced by 50% following co-administration of DEX with docetaxel (*P*<0.05) (Figure 5C).

**Figure 6:**
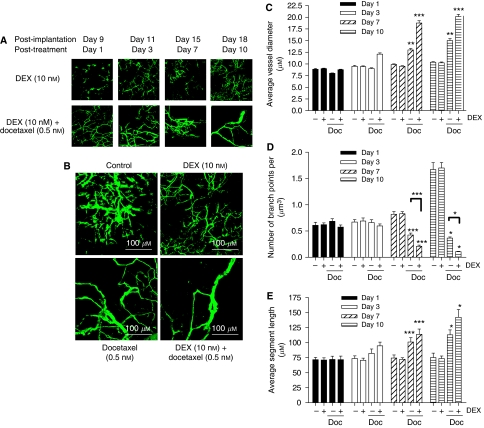
Enhancement of the antiangiogenic effect of docetaxel by combination with dexamethasone (DEX) in a dorsal skin flap assay. PC3 tumour fragments were implanted in a subcutaneous ‘window chamber’ apparatus on the backs of SCID mice. The development of intratumoural blood vessels was imaged over a 10-day period following treatment with DEX or docetaxel (Doc). The vasculature was visualised using a multiphoton confocal microscope following a tail vein injection of 50 *μ*l of 150 kDa FITC-labeled dextran (50 mg ml^−1^). (**A**) Representative images showing the response of blood vessel in PC3 tumours receiving 10 nM DEX (top panel) or combined treatment with 10 nM DEX plus 0.5 nM Doc. Images clearly depict a reduction in blood vessels in tumours receiving Doc. (**B**) Representative images showing the comparison of the vasculature within PC3 tumours 10-day post-initiation of treatment administration. Images shown are for vehicle control (top left quadrant), 10 nM DEX (top right quadrant), 0.5 nM Doc (lower left quadrant) and the combined treatment of 10 nM DEX plus 0.5 nM Doc (lower right quadrant). The loss of small vessels is again evident in the Doc-treated tumours with further selection for large diameter vessels in those tumours receiving the combined DEX/Doc treatment. (**C**) Quantitative analysis of vessel diameter in vehicle control-, 10 nM DEX-, 0.5 nM Doc and 10 nM DEX/0.5 nM Doc-treated PC3 tumours at four different times post-initiation of treatment. Bars shown represent the mean±s.e.m. value calculated from the analysis of 10 independent fields in each of five mice. (**D**) As in (**C**) except that bars represent the mean±s.e.m. number of vessel branch points determined in the tumours. (**E**) As in (**C**) except that bars represent the mean±s.e.m. value calculated for vessel segment length determined in the tumours. Statistically significant differences in vessel diameter, number of branch points or vessel segment length were determined using a one-way ANOVA. ^*^P<0.05; ^**^P<0.01; ^***^P<0.001.

The antiangiogenic effect of DEX and docetaxel was studied further, exploiting a chamber assay in the skin flap of SCID mice to determine the effect of these agents upon the vascularisation of an implanted fragment of a PC3 tumour. The parameters of vessel diameter, branch points and segment length were measured from images taken of the growing tumours at days 9, 11, 15 and 18 post-implantation (drug treatments initiated on day 8). Images shown compare the effect of DEX on tumour vasculature against the potent antiangiogenic effect of co-administering DEX with docetaxel over the 10-day period (Figure 6A). A comparison of the effect of vehicle control, DEX, docetaxel and the combined DEX/docetaxel treatment on the organisation of the tumour vasculature at day 10 post-treatment is also shown (Figure 6B).

Images of the tumour vasculature were subjected to further quantitative analysis. Treatment with DEX alone resulted in no significant difference in vessel diameter (Figure 6C), the number of branch points observed (Figure 6D) or the average segment length of the blood vessel (Figure 6E) relative to control over the course of the experiment. In contrast, administration of docetaxel resulted in a marked reduction in the number of small diameter blood vessels that could be observed throughout the tumour mass 7 (*P*=0.018) and 10 days (*P*=0.011) post-treatment relative to control (Figure 6C). This coincided with a decrease in the number of vessel branch points detected (Figure 6D) and an increase in the vessel segment length (Figure 6E) measured within the tumours on each of these days.

The combination of DEX with docetaxel enhanced the antiangiogenic response further, potentiating the increase in the average vessel diameter and vessel segment length within the tumour and reducing further the number of vessel branch points detected, relative to the effect of docetaxel alone, 7 and 10 days post-treatment. This was particularly evident in quantifying the number of branch points where statistically significant differences between the DEX/docetaxel and docetaxel treatments were observed at days 7 (*P*<0.001) and 10 (*P*<0.05). With respect to the other parameters under investigation, the trend towards an increase in the vessel diameter and segment length in the DEX/docetaxel-treated tumours relative to docetaxel alone approached but did not attain statistical significance in a two-tailed *t*-test in this experimental design (e.g., *P*=0.06 for difference in segment length and diameter at day 10). Furthermore, although again not statistically significant, our results suggest that the combination of DEX with docetaxel increases the rate of onset of the observed antivascular effect in the tumours, supported by the detection of an increased average vessel diameter and average segment length within 3 days of treatment.

## Discussion

Docetaxel is at present the chemotherapy agent of choice in the treatment of metastatic CRPC. To offset the risk of developing docetaxel-induced hypersensitivity, patients treated with docetaxel also received DEX pre-medication (Tannock *et al*, 2004). As DEX administration alone has been reported to have clinical benefit in CRPC patients (Venkitaraman *et al*, 2008), this has led to speculation that DEX may contribute in part to the increased survival benefit of patients receiving docetaxel. The objective of our study was to characterise a mechanism that may explain a synergy by which DEX may potentiate the clinical benefit of docetaxel in patients with CRPC.

Experiments were conducted primarily on the representative CRPC cell line PC3 (originally derived from a bony metastasis of human prostate carcinoma) with key experiments repeated in a transformed bone marrow endothelial cell line, as outgrowth of secondary lesions within the bone is the predominant complication associated with CRPC. Consistent with its anti-inflammatory action, we observed that DEX administration decreased the constitutive NF-*κ*B transcriptional activity in the PC3 cells. Consequently, we were able to show a decreased transcription and/or secretion of the CXC-chemokines IL-8 and CXCL1 and the proangiogenic growth factor, VEGF. This response was also observed in hBMECs, where DEX treatment resulted in the suppression of IL-8 and CXCL1 gene transcription and/or secretion.

Constitutive activity of NF-*κ*B has been reported in CaP cell lines and/or biopsy tissue and is associated with regulating the expression of genes that induce cell proliferation and/or underpin cell survival (Huang *et al*, 2001; Fradet *et al*, 2004; Ross *et al*, 2004; Shukla *et al*, 2004; Sweeney *et al*, 2004; Domingo-Domenech *et al*, 2005). However, despite suppressing the constitutive activity of the NF-*κ*B transcription factor, DEX administration failed to reduce the proliferation or the viability of PC3 tumour cells or hBMECs. In addition, we observed that co- or pre-administration of DEX failed to potentiate the cytotoxicity of docetaxel in PC3 cells. Interestingly, our findings support several previous observations that report the absence of a cytotoxic or antiproliferative effect of DEX in CaP cells. For example, DEX has been reported to antagonise rather than enhance the potency of conventional cytotoxic chemotherapy agents, including doxorubicin, cisplatin or etoposide, in PC3 cells (Carollo *et al*, 1998). Indeed, DEX has been shown to induce resistance to several cytotoxic agents, including the taxane, paclitaxel, in cells isolated from surgical resections of prostate tumours (Zhang *et al*, 2006) and to a range of cytotoxics in xenograft models of CaP (Zhang *et al*, 2007). Studies in macrophages, endothelial, airway smooth muscle and cancer cells have shown that DEX-induced glucocorticoid receptor signalling increases the expression of MAPK phosphatase-1, resulting in decreased MAPK signalling in these cells and contributing to the anti-inflammatory effect (Wu *et al*, 2005; Abraham *et al*, 2006; Furst *et al*, 2007; Issa *et al*, 2007). Interestingly, overexpression of MAPK phosphatase-1 has been shown to attenuate taxane-induced, proapoptotic JNK signalling in breast cancer cells (Wu *et al*, 2005). Therefore, this suggests a possible mechanism to explain why DEX fails to enhance the sensitivity of CaP or endothelial cells to docetaxel-induced cytotoxicity.

Taxane administration has previously been reported to induce the expression of the proangiogenic CXC chemokine IL-8 in a range of cancer cell lines (Collins *et al*, 2000; Uslu *et al*, 2005). We too have observed this effect in PC3 cells and hBMECs. Docetaxel administration potentiated the transcriptional activity of AP-1 and NF-*κ*B in PC3 cells and hBMECs, resulting in increased synthesis and secretion of IL-8 and a related CXC-chemokine CXCL1. However, co-administration of DEX was observed to attenuate the docetaxel-induced AP-1 and NF-*κ*B transcriptional activity and abrogated docetaxel-induced CXC-chemokine gene transcription and secretion in PC3 cells and endothelial cells. We have recently reported that the induction of IL-8 signalling in hypoxic and normoxic CaP cell lines activates cell survival signalling and confers a survival advantage to these cells in response to DNA-damage agents (Maxwell *et al*, 2007; Wilson *et al*, 2008b), antiandrogens (Seaton *et al*, 2008) and biological inducers of apoptosis such as TNF-receptor apoptosis-inducing ligand (Wilson *et al*, 2008a). Direct inhibition of NF-*κ*B using BAY11-7082 or direct antagonism of IL-8 signalling, targeting the ligand with a neutralising antibody or through a CXCR2-receptor antagonist, failed to potentiate the cytotoxicity of docetaxel in these cells. It has been previously reported that overexpression of IL-8 in ovarian cancer cells had no appreciable effect on paclitaxel resistance *in vitro* (Duan *et al*, 2002). However, recent *in vivo* studies using liposome-encapsulated siRNA oligonucleotides to target IL-8 expression has shown that targeting this chemokine reduces microvessel density and significantly enhances the antiproliferative effect of docetaxel on HeyA8 and SKOV3ip1 ovarian tumour growth (Merritt *et al*, 2008).

Dexamethasone has been shown to suppress proangiogenic gene transcription and secretion in a range of CRPC cells *in vitro*, whereas a subcutaneous administration of DEX inhibits tumour growth and angiogenesis in an *in vivo* DU145 xenograft model of CRPC (Yano *et al*, 2006). The impact of co-administering DEX on the antiangiogenic activity of docetaxel was initially studied using an *in vitro* assay that measures the capacity of endothelial cells to promote vessel development in a co-culture system. Dexamethasone had a minimal but not statistically significant effect in reducing the development of CD31+ vessels. However, addition of DEX did potentiate the pronounced antiangiogenic activity of docetaxel, reducing both the observed mean vessel length and mean vessel area. This result was further confirmed through real-time imaging of the vasculature in an implanted PC3 tumour in an SCID mouse, showing a significant loss of intratumoural vascular development in tumours that were concurrently treated with DEX and docetaxel. A pronounced decrease in vessel branching and a reduction in the number of small developing vessels within the tumour was clearly visible within the tumour, consistent with the reduced capacity of the tumour to stimulate *de novo* vessel development. Therefore, the ability of DEX to suppress IL-8, CXCL1 and VEGF would appear to underpin an enhanced suppression of the tumour vasculature in the PC3 model of CRPC and potentiate the antiangiogenic effect of docetaxel. Consequently, using either DEX to inhibit IL-8 gene expression in CRPC cells or the IL-8 gene-silencing strategy in ovarian cancer cells (Merritt *et al*, 2008) indicates that IL-8 signalling does not modulate the mechanistic basis of taxane-induced cell death in tumour cells. Instead, these studies both suggest that suppressing the level of IL-8 signalling capacity within the microenvironment of the tumour clearly enhances the therapeutic benefit of taxanes, mediated through a pronounced suppression of angiogenesis within the tumour. Although these studies have not delineated the mechanism underpinning the reduced vascularisation observed in the DEX/docetaxel-treated tumours, the apparent absence of an effect in regulating endothelial cell survival suggests that the decrease in CXC-chemokine signalling potential within the tumour may attenuate the capacity of endothelial cells to migrate and/or promote tube formation. Understanding the mechanism of action will be the subject of future experiments in our laboratory.

In conclusion, we have shown that DEX attenuates docetaxel-induced AP-1 and NF-*κ*B activation, reducing the synthesis of proangiogenic factors in both CaP and endothelial cells, with an ultimate effect of reducing *de novo* vessel development in a prostate tumour. Our data indicate that DEX potentiates the antiangiogenic as opposed to the cytotoxic activity of docetaxel on tumour cells. Angiogenesis is critical in accelerating primary tumour growth and in establishing colonisation at secondary sites and therefore, may explain why a combination of these two agents extends the median survival of patients with advanced CaP. Consequently, many of the novel antiangiogenic agents undergoing clinical trials may find significant utility and purpose in treating CRPC, especially in combination with existing agents such as docetaxel.
